# Subsecond Terahertz Spectroscopic Mapping of Corneal Hydration Gradients

**DOI:** 10.21203/rs.3.rs-11271533/v1

**Published:** 2026-10-07

**Authors:** Zachery B. Harris, Arash Karimi, Sarah Weissbart, Robert Honkanen, M. Hassan Arbab

**Affiliations:** 1 Dept. of Biomedical Engineering, Stony Brook University, Stony Brook, NY, USA, 11794; 2 Dept. of Ophthalmology, Renaissance School of Medicine, Stony Brook University, Stony Brook, NY, USA, 11794

## Abstract

Terahertz time-domain spectroscopy (THz-TDS) is sensitive to tissue water content, but slow acquisition and stringent alignment requirements have hindered its *in vivo* applications, particularly in corneal imaging. We present a compact spherical-scanning THz imaging system that acquires 100-ps time-domain waveforms at a rate of 2 kHz and generates a 10 × 10-pixel image over a 40° × 40° field of view in 0.8 s. Time-of-arrival measurements across the image are used to estimate and automatically correct lateral and axial misalignments. The system was evaluated using a drying contact-lens corneal phantom and seven *ex vivo* porcine eye globes while sample mass was recorded continuously. In both models, the reflected THz signal exhibited strong correlations with gravimetrically measured hydration changes. Sparse deconvolution of the THz pulses reflected from the contact lens separated echoes from the anterior and posterior interfaces, enabling simultaneous mapping of interfacial reflectivity and optical thickness. The longitudinal sample hydration and optical thickness maps also revealed complementary spatially heterogeneous drying patterns, potentially indicating a limitation of using thickness as a surrogate for hydration evaluation in clinical practice. Finally, we evaluated the sensitivity of THz spectroscopy to hydration change in *ex vivo* eye globes and found a median 0.7% decrease in THz reflectivity per 1% hydration loss. These results demonstrate rapid, motion-tolerant THz spectral imaging of corneal-scale spherical surfaces and support its further development for noncontact *in vivo* assessment of corneal hydration.

## 1. Introduction

The cornea derives its transparency and refractive power from the highly organized collagen architecture of the stroma, which constitutes approximately 90% of the corneal thickness. Stromal organization and fibril spacing are hydration dependent and are maintained by the barrier and pump functions of the endothelium [1–7]. When corneal deturgescence is disrupted, stromal water content rises, increasing light scattering and impairing vision. Clinically, hydration is commonly inferred from central corneal thickness (CCT) measured by ultrasound pachymetry or optical coherence tomography (OCT). CCT, however, is an indirect surrogate for water content and is affected by physiologic intraindividual and interindividual variability [8,9]. A single central thickness measurement is also insensitive to rapid changes and spatially heterogeneous or subclinical edema associated with Fuchs endothelial corneal dystrophy (FECD) [10–14], other endothelial disorders, contact-lens wear [12,15], and surgical trauma [16,17].

Several optical approaches have been investigated for assessing corneal hydration. Confocal Raman spectroscopy estimates the relative water and protein content of the tissue by comparing the intensities of the O-H and C-H vibrational bands; however the required optical power exposure raised ocular safety concerns [18]. Differential absorption OCT compares two wavelengths of light, one inside a water absorption band and one outside, to estimate tissue water content, but its accuracy can be affected by scattering and chromatic dispersion [19]. Near-infrared spectroscopy has been demonstrated for single-point measurement of *ex vivo* stromal water content, but has not yet been extended to spatially resolved hydration mapping [20]. Brillouin microscopy measures a hydration-sensitive mechanical response and serves only as an indirect assessment of hydration [21]. Despite their potential, none of these methods have achieved widespread clinical adoption for corneal hydration assessment.

Liquid water strongly absorbs terahertz (THz) radiation across the 0.1–3 THz band, making THz spectroscopy intrinsically sensitive to changes in tissue hydration, and thus motivating the application of THz imaging to several biomedical applications [22–27]. Furthermore, THz time-domain spectroscopy (THz-TDS) provides insight into sample geometry through time-of-flight measurements [28–30]. These characteristics make THz-TDS reflection imaging well suited for non-contact monitoring of corneal hydration. Prior studies have characterized corneal sublayers, dehydration, *in vivo* sensing, and hydration changes associated with intraocular pressure or endothelial injury [31–35]. Translating this sensitivity into a practical *in vivo* imaging system, however, has been hindered by substantial technical challenges. Careful alignment is required to provide a strong back-reflected THz signal as the corneal radius of curvature (approximately 7–8 mm) is similar in scale to THz wavelengths (300 micrometers to 1 mm) as well as the millimeter-scale focused beam waists, and depth of field used for imaging. Non-contact single-point measurements of corneal properties at normal incidence can be achieved by matching the phase front of the THz light to the spherical sample surface [36–38]. Forming an image requires a system to maintain this alignment as the sample is illuminated from different directions with normal incidence angle across the three-dimensional surface of the cornea. Aligning the surface’s center of curvature to the focus of a large-aperture off-axis parabolic mirror [39–41] or custom-designed lens [42] maintains the phase-front-matching condition at the surface as the beam is translated across the aperture. Architectures using a pair of identical elements improve the acquisition speed by restricting mechanical scanning motion to rotations of a gimballed mirror [43]. We recently used this approach to demonstrate a THz imaging system that captures images in 3–4 seconds with topographical surface accuracy of 20μm(~λ/40) at the peak frequency of the scanner [42,44]. However, acquisition times of even a few seconds are susceptible to artifacts from involuntary eye motion during *in vivo* scans, constraining the available time for both alignment and image acquisition.

We present a compact spherical-scanning system that acquires 100-ps THz-TDS waveforms at kilohertz rates to enable sub-second spectral imaging of the corneal surface. In the following section, we give a brief overview of the system design and describe how we use time-of-flight measurements for automated alignment and motion compensation. Sections 3 and 4 present THz images from drying experiments using a contact-lens phantom and *ex vivo* porcine eye globes, respectively, and show that in both cases, there is very good agreement to hydration calculated from simultaneous gravimetric measurement. Finally, in Section 5 we compare the implications of the two experiments and discuss how these techniques will be applied in the future *in vivo* measurements. We will present preliminary results indicating potential sensitivity limitation of THz measurement to hydration change in phantoms and *ex vivo* corneas.

## 2. High-speed THz corneal imaging system

Figure 1(a) shows the schematic of the spherical scanning system. This design is based on our previously reported architectures consisting of a stationary transceiver system, a motorized gimballed mirror, and a two-lens focusing system [42,44] with the addition of an automatic sample re-alignment capability. A transceiver section focuses a THz beam onto the gimballed mirror, which steers the diverging reflection across the lens pair. The custom-designed hyperbolic-elliptic lens pair collimates and refocuses the beam to different positions on the spherical target while maintaining the phase-front-matching properties across the field of view (FOV). At each scan position within the images, we record the full 100 picosecond time-domain electric field reflected from the sample.

Two factors limited the imaging speed of previous systems: waveform acquisition and beam steering. Earlier implementations used asynchronous optical sampling (ASOPS) to record 100 waveforms/s. Reconfiguring the system for electronically controlled optical sampling (ECOPS) increased the acquisition rate to 2,000 waveforms/s [45,46]. To complement the increase in data acquisition, we also upgraded the beam steering system. The rendering of the scanner in Fig. 1(b) shows the arrangement of various motors in the setup. Earlier versions employed a compact gimbal originally developed for our THz PHASR scanners designed for imaging planar targets [45]. The gimbal was formed by stacking a goniometer on a rotation stage, as shown in Fig. 1(c). The motors in these two stages drove the desired axes of motion of the scanning mirror platform through different gearing systems in a low-profile package. The resulting gimbal provided speeds of up to 208°/s along the elevation axis (goniometer) and 300°/s along the azimuthal scanning axis (rotation stage). The mechanical limits of the goniometer constrained the FOV in the elevation direction to ±20°. The updated design, shown in Fig. 1(d), maintains the same heliostat geometry but eliminates the geared stages and instead drives the rotations directly. This improves the speed of the mirror rotation to 18,000°/s along the azimuthal and elevation directions. Consequently, a 40°×40° scan (about 6 mm × 6 mm on the surface of a human cornea), which previously required ~4 s can now be completed in 0.8 s. Furthermore, the scan range is now only limited by the lens aperture size.

### 2.1 Automated realignment for tracking eye motion

For *in vivo* scanning, reliable initial alignment and rapid recovery from misalignment are essential due to the stringent phase-front-matching requirements of the imaging geometry. Figure 1(b) shows the scanner mounted on three motorized linear stages for positioning relative to the target along the $\overset{ˆ}{x},\overset{ˆ}{y}$, and $\overset{ˆ}{z}$ axes. Lateral alignment in the $\overset{ˆ}{x}$ and $\overset{ˆ}{y}$ directions is achieved by translating the entire imaging system to center the target on the optical axis the lens pair. In contrast, axial alignment along $\overset{ˆ}{z}$ is adjusted by translating only the final lens along the optical axis, as indicated by the dashed green box in Fig. 1(a). A cage assembly maintains alignment between the lenses and the gimballed mirror during lens translation. This configuration reduces the required motor size and preserves clearance between the scanner and the patient or sample. Alignment can be evaluated based on well-defined patterns of the time of arrival (TOA) of the THz pulses across the FOV. Following initial coarse positioning, or after subject motion, the system can estimate and iteratively auto-correct the alignment offsets.

The use of TOA for estimating the alignment follows directly from the definition of the scanning coordinates centered at the system focus illustrated in Fig. 2(a)–(b). The red shaded region represents the full scanning FOV of the scanner. It is convenient to describe the scan in a spherical coordinate system, where

(1) $$\begin{array}{c} x=\rho \;\text{sin}\;\theta \;\text{cos}\;\phi ,\\ y=\rho \;\text{sin}\;\theta \;\text{sin}\;\phi ,\\ z=\rho \;\text{cos}\;\theta . \end{array}$$

In this coordinate system, each pixel signifies an angular coordinate position, (θ,ϕ), and the radial coordinate, ρ, relates to positions along the time axis of the THz signal. When a sphere with radius $\rho_{S}$ is well aligned, the THz pulses of all pixels will have a constant TOA corresponding to surface height $\rho (\phi ,\theta )=\rho_{S}$, shown as the blue line in Fig. 2(b). However, in the more general case when the sphere is offset by $x_{0},y_{0}$, and $z_{0}$ in the $\overset{ˆ}{x},\overset{ˆ}{y}$, and $\overset{ˆ}{z}$ directions, respectively, the surface of a sphere is given by the equation

(2) $$\rho_{S}^{2}={\left(x-x_{0}\right)}^{2}+{\left(y-y_{0}\right)}^{2}+{\left(z-z_{0}\right)}^{2}.$$

From the symmetry of the scanning geometry, it is apparent that there are two types of offset: lateral offsets in the $\overset{ˆ}{x}$ and $\overset{ˆ}{y}$ directions as shown in Fig. 2(c) and axial offsets in $\overset{ˆ}{z}$ as shown in Fig. 2(d). Expanding Eq. (2) in terms of Eq. (1) and solving for ρ then provides the equation for the surface in terms of the offsets and angular coordinates,

(3) $$\rho =\sqrt{\rho_{S}^{2}-{\left(x_{0}\;\text{sin}\;\phi -y_{0}\;\text{cos}\;\phi \right)}^{2}-{\left(\left(x_{0}\;\text{cos}\;\phi +y_{0}\;\text{sin}\;\phi \right)\text{cos}\;\theta -z_{0}\;\text{sin}\;\theta \right)}^{2}}+\left(\left(x_{0}\;\text{cos}\;\phi +y_{0}\;\text{sin}\;\phi \right)\text{sin}\;\theta +z_{0}\;\text{cos}\;\theta \right).$$

Along the direction of the lateral offset, $\phi_{0}=\text{atan}\left(y_{0}/x_{0}\right)$,

$$x=r\;\text{cos}\;\phi_{0},\;y=r\;\text{sin}\;\phi_{0},\;r=\rho \;\text{sin}\;\theta ,\;r_{0}^{2}=x_{0}^{2}+y_{0}^{2},$$

and the contribution of the lateral components of the surface can be simply expressed as

$$\begin{array}{l} \left(x_{0}\;\text{cos}\;\phi_{0}+y_{0}\;\text{sin}\;\phi_{0}\right)=r_{0},\\ \left(x_{0}\;\text{sin}\;\phi_{0}-y_{0}\;\text{cos}\;\phi_{0}\right)=0. \end{array}$$

The surface equation can then be expanded to express the surface height along $\overset{ˆ}{r}$ in terms of only the θ coordinate,

(4) $$\rho =\sqrt{\rho_{S}^{2}-{\left(r_{0}\;\text{cos}\;\theta -z_{0}\;\text{sin}\;\theta \right)}^{2}}+r_{0}\;\text{sin}\;\theta +z_{0}\;\text{cos}\;\theta .$$

When $r_{0}$ and $z_{0}$ are small compared to $\rho_{S}$, the radical can be approximated by simply $\rho_{S}$. However, our THz system does not provide an absolute measurement of the TOA. Therefore, the measured surface height, Δρ, with respect to the position at the center of the image, ρ(0), is given by

(5) $$\Delta \rho =\rho \left(\theta \right)-\rho \left(0\right)=\left(\rho_{S}-\rho \left(0\right)\right)+r_{0}\;\text{sin}\;\theta +z_{0}\;cos\;\theta .$$

Thus, the surface profile is measured with respect to a sphere with radius $\left(\rho_{S}-\rho (0)\right)$ centered at the origin, with Δρ=0 at all pixels for a perfectly aligned sphere of any size. This is shown in Fig. 2(c) and (d) by the difference between the solid blue line, which represents ρ, and the dashed blue line representing a sphere with radius ρ(0) centered on the focus. Qualitatively, it can be seen from Eq. 5 or Fig. 2(c) and (d) that for a lateral misalignment, we expect to measure an increasing surface “height” in the direction of misalignment. In contrast, axial misalignments produce a peak or valley when the sphere is too close or too far, respectively. To estimate the required correction, we used the second order Maclaurin expansion of Eq. 5 with respect to θ,

(6) $$\Delta \rho =\left(\rho_{S}+z_{0}-\rho (0)\right)+r_{0}\theta -\frac{z_{0}\theta^{2}}{2}.$$

While we cannot measure the constant offset in parentheses in Eq. 6, lateral misalignments result in a roughly planar surface profile, whereas axial misalignments produce paraboloidal profiles. The measured surface heights for a sphere under different misalignment conditions shown in Figure 3 demonstrate such cases. We estimate the movement needed to align the system by fitting a paraboloid to Δρ(x,y). The components, $x_{0}$ and $y_{0}$ of $r_{0}$ define a plane, shown in Fig. 3(a), which can be used to determine the lateral direction to move. The full parabolic fit is demonstrated by the blue surfaces in Fig. 3(b). Since only a subset of the full imaging points are needed to fit the surface, we use rapid scans such as line scans along each of the two motor axes to reduce the alignment time.

### 2.2 Removal of the System Background Signal

The recorded THz-TDS signals have substantial background contributions from internal reflections within the collocated transceiver, electronic noise, and other instrumental sources. Additionally, during measurements that last many hours, there is an appreciable drift of the TDS acquisition period. The background signal is primarily composed of low-frequency components below 0.1 THz and does not change from pixel to pixel. Therefore, this drifting background signal is used to align the TDS measurements [47], as shown in Fig. 4.

Before each imaging session, two images are acquired: a “reference” image of a metal sphere and a “background” image taken without a sample in place. Representative traces from these images, together with an example sample trace, are shown in Fig. 4(a). A double-Gaussian bandpass filter is applied to each signal to isolate the low-frequency baseline below 0.1 THz and suppress high-frequency noise above 1 THz. The low-frequency components, shown in Fig. 4(b), are similar from image to image and capture the drift of the system. On the other hand, the mid-frequency components, in Fig. 4(c), retain the information about the targets. The low-frequency components are aligned using cross-correlation, as shown in Fig. 4(d), and the resulting time shift is also applied to the corresponding mid-frequency signals in Fig. 4(e). The alignment is performed across all pixels to ensure accurate time-of-flight measurements across the FOV. After this correction, the remaining timing differences between the reference and sample reflections are due to changes in radius or position of the metal and sample spheres.

Residual background contributions within the passband of the double-Gaussian filter are given by the average time-domain trace over all pixels of the background image, shown in Fig. 4(f). This baseline signal is subtracted from the reference and sample data, as shown in Fig. 4(g). Finally, a split Blackman window is applied to isolate the relevant reflections and taper the edges of the selection. The resulting reference and sample waveforms, shown in Fig. 4(h), are then used in target-specific processing, which will be discussed in the following sections.

## 3. Terahertz Spectral Mapping of Hydration Gradients and Sample Thickness in Corneal Phantoms

We performed initial system validation using a corneal phantom, comprising a disposable contact lens mounted on a metal sphere, as depicted in Fig. 5(a). The sphere was mounted on an analytical balance to continuously record mass loss while the contact lens dried under ambient conditions, providing an independent ground truth measurement of the bulk water content. After aligning the scanner to the bare sample holder sphere, the contact lens was transferred from a hydrating solution to the sphere at t=0min and THz images were acquired once per minute. The lens hydration was calculated by

(7) $$[\text{Hydration}\;\%]=100\times \frac{m(t)-m_{\text{dry}}}{m_{0}},$$

where m(t) is the mass of the contact lens at time $t,m_{0}$ is the initial hydrated mass of the contact lens, and $m_{\text{dry}}$ is the mass of the contact lens after it was allowed to dry completely. Figure 5(b) illustrates the change in hydration over the drying process. The initial hydration of the lens, 72%, differs only slightly from the manufacturer’s specified value of 69%. This 3% variation is consistent with previous measurements of hydrogel contacts made by our group and others [48–50]. Our laboratory is maintained at approximately 23°C and humidity levels were stable during these experiments at about 30% relative humidity. Over 15 minutes of measurement, the lens hydration decreased to 16%. Peak-to-peak THz amplitude images acquired at the start and end of this period are shown in Fig. 5(c) and (d), and the mean time-domain waveforms from a region of interest (ROI; dashed boxes) are shown in Fig. 5(e). As the lens dried, the amplitude of the front (air-lens) reflection decreased while the rear (lens-metal) reflection amplitude increased and its delay from the first reflection decreased. This shift in delay results from changes in both the thickness and the effective refractive index of the hydrogel material as its relative water content decreased.

As the dehydration progressed, the central region of the lens became too thin to separate the front and rear reflections using time-windowing alone. Consequently, the contact lens reflectivity appears to increase in the center of the image due to the strong reflection from the metal substrate, regardless of time-window used to calculate the peak-to-peak amplitude. Since this artifact is not representative of a biological sample, we used a ROI adjacent to the center where the lens remained thicker to illustrate the behavior of the sample more clearly. Computing the deconvolution of the reflected signal by a known reference pulse yields the impulse response of the sample, in which the two reflections can be separated. Since the sample is made of three distinct layers with well-defined boundaries, we expect that the impulse response function will contain a small number of peaks which each correspond to a reflection at one of the interfaces. Therefore, we used a time-domain sparse deconvolution method to separate the reflections [30,51]. In brief, the measured signal, $E_{\text{meas}}$, is assumed to be the result of convolving a reference time-domain signal, $E_{\text{ref}}$, with an impulse response function, h, in the presence of noise, ϵ. That is, for each point n, in the measured time-domain trace, $E_{\text{meas}}[n]=h[n]*E_{\text{ref}}[n]+\epsilon [n]$, or in matrix form

(8) $$E_{\text{meas}}={\text{E}}_{\text{ref}}h+\epsilon ,$$

where the rows of the matrix ${\text{E}}_{\text{ref}}$ are composed of delayed and time-reversed copies of the reference signal. The impulse response h is estimated by minimizing the loss function

(9) $$ℒ=\frac{1}{2}{\left\|{\text{E}}_{\text{ref}}h-E_{\text{meas}}\right\|}_{2}^{2}+\lambda \|h{\|}_{p},$$

where the first term is the difference between the measured and calculated signals, and the second term enforces sparsity of h when p is either 0 or 1. The regularization parameter λ controls the balance between the sparsity and goodness of fit. We implemented an iterative shrinkage method with soft thresholding to find the h which minimizes ℒ for each trace [52]. Figure 5(f) shows the deconvolved traces of the image ROIs. Two distinct peaks, $h\left(\tau_{1}\right)$ and $h\left(\tau_{2}\right)$ can be observed, corresponding to the air-lens and lens-metal interfaces, respectively. Deconvolution also reduces the ambiguity of the TOA of these peaks, $\tau_{1}$ and $\tau_{2}$, compared to the original electric field measurements. Their difference, $\Delta \tau =\tau_{2}-\tau_{1}$, provides a measurement of the round-trip optical thickness of the sample.

Figure 6(a) shows that both $h\left(\tau_{1}\right)$ and $h\left(\tau_{2}\right)$ varied approximately linearly with hydration within the ROI. The positive slope of $h\left(\tau_{1}\right)$ illustrates the direct dependence of the air-lens reflection on the hydration of the sample, while the negative slope of $h\left(\tau_{2}\right)$ demonstrates that at higher hydrations, less of the energy of the pulse reaches and reflects from the metal surface of the sphere. Both reflections show strong linearity with coefficients of determination of $R^{2}\approx 0.9$. The delay, Δτ, is affected by both the swelling and changes in refractive index of the lens and is plotted in Fig. 6(b). This metric shows a stronger correlation to the hydration, $R^{2}=0.96$.

A key advantage of the sparse deconvolution is that the two reflections in the impulse responses remain sufficiently separated to be independently analyzed over the full image. Figure 7(a) shows maps of $h\left(\tau_{1}\right)$ throughout the drying process. Unlike the peak-to-peak images, these images do not show an apparent increase in reflectivity at the center of the image due to the influence of the lens-metal reflection. Instead, the amplitude of the pulse decreased relatively uniformly over the image as the lens dries. Some regions, including the location of the ROI analyzed in Fig. 5, have persistently higher amplitude than the surrounding areas, possibly indicating higher than average local water concentration. Other nearby regions, such as just above and below the center of the image, also initially show high reflection, but converge to the same levels as their surroundings by the end of the experiment. This suggests the diffusion of water along local hydration gradients within the lens. Maps of Δτ are shown in Fig. 7(b) and capture the profile of the lens that results in its negative power: it is thinner in the middle of the lens and thicker at the edges. Although Δτ decreases across the drying time, the spatial pattern remains consistent, indicating that the lens profile was similar between hydrated and dry states. Notably, the distributions observed for the amplitude and Δτ metrics differ in both their initial patterns and how they evolve during the drying process, indicating that the two metrics provide complementary information about the sample. This distinction is highlighted in Fig. 7 by overlaying maps of the spatial gradient on each of the images. Such spatial variations are not captured by hydration measurements, which are derived from sample’s bulk mass and represent an average value. These results therefore emphasize the advantages of imaging, which characterizes the local variations of the samples.

## 4. Terahertz Spectral Mapping of *ex vivo* Eye Globe Hydration Gradients

Measurement of *ex vivo* samples followed a similar procedure to that of the phantoms. Enucleated eye globes were obtained following the conclusion of other porcine studies at the Stony Brook University’s Division of Laboratory Animal Resources (DLAR). The *ex vivo* samples became available in two batches. Eyes A-C were measured in March 2026, when the relative humidity in the laboratory ranged between 15% and 40%, while for samples D-G (June 2026) the lab conditions were more humid, with relative humidity between 40% and 50%. Sensor failure during eyes B and C led to incomplete recording of the humidity during those experiments. The ambient temperature was constant for both cohorts at 23°C. The globes were placed in a 3D-printed sample holder mounted on the analytical balance as shown in the inset in Fig. 8(a). We allowed the eyes to dry while continuously recording mass measurements. THz-TDS images were acquired approximately every 4 minutes until the automatic alignment could no longer resolve an adequate surface for alignment, due to the change in shape of the sample. For all samples, the mass continued to decrease after imaging concluded, indicating that the fully dry mass of the tissue had not been reached. We therefore calculated the hydration loss during the experiment by

(10) $$[\text{Hydration}\;\text{Loss}]=100\%\times \frac{m_{0}-m(t)}{m_{0}}.$$

Table I summarizes the total imaging duration, average laboratory humidity, initial and final masses and final hydration loss for each of the samples.

Here we use sample G as a representative example because it provided the longest continuous measurement interval. Figure 8(a) shows the decrease in mass as water evaporated from the ocular surface. Figure 8(b) shows the corresponding evolution of the time-domain traces from a small ROI at the center of the image together with a reference pulse from the same area of a metal sphere of similar radius (black dashed line). The increase in TOA of the reflected pulse over drying time is caused by dehydration-induced deformation of the eye and the resulting bulk motion within the mount. This motion was distinctly different for each eye sample; however, the automatic alignment system maintained the proper focal position throughout the measurements. Unlike the contact lens measurements, no reflection from the rear surface of the tissue can be seen in the time-domain waveforms. Consequently, the optical thickness cannot be measured.

Figure 8(c) and (d) show the evolution of peak-to-peak THz images as the eye globe dried. In each plot the peak-to-peak values are normalized pixel-by-pixel by those of the metal reference image. The THz images in Fig. 8(c) show the early drying period while those in Fig. 8(d) show the hydration state at five-hour intervals at the end of the experiment. Beyond the clear decrease in overall amplitude, the local variations indicate spatial differences which are not captured by the mass measurements. For instance, the earlier images show a slight gradient of reflectivity from the lower left to the upper right. However, after 5 hours of drying this spatial pattern resolved into two separate regions of reflectivity. Starting at about 8 hours, a band of lower reflectivity forms in the image, crossing from the upper left to lower right of the image. The band persists until approximately 20 hours, and after 24 hours it is no longer distinct as the surrounding areas have decreased to similar levels.

As observed in the contact lens imaging experiment, spatial variations indicate that point measurements may not directly reflect the bulk hydration of the tissue. Similar persistent spatial features were present in the peak-to-peak amplitude maps for all seven *ex vivo* eye globes, though the pattern varied by sample. Nevertheless, the comparisons between the mean relative peak-to-peak amplitude and the hydration loss, shown in Fig. 9(a)–(g), demonstrate that the THz imaging consistently captures the effect of the evaporation. Linear fits to the data (solid lines) have coefficients of determination ranging from $R^{2}=0.77$ to 0.91 with the only sample falling below $R^{2}=0.80$ being the one that exhibited the smallest hydration loss. The dashed lines show the 95% prediction interval (i.e., α=0.05) for the measured peak-to-peak value at any hydration value [53,54].

Potential differences in initial hydration prevent direct pooling of data across eyes into a single regression. Examination of the individual regressions, however, still provides insight into the THz system’s sensitivity to hydration change. As shown in Fig. 9(h), the regression slopes indicate that a 1% hydration loss corresponds to a 0.4% to 1% decrease in normalized peak-to-peak amplitude, with a median of 0.7% decrease per 1% hydration loss. For practical applications, it is important to estimate the range of hydration values that might correspond to a measured peak-to-peak value. Inverse regression, sometimes called calibration, provides an estimate for the 95% calibration confidence interval for the true value of hydration. This corresponds to the width of the prediction band at the measured peak-to-peak value [53,55]. As these widths are approximately constant over the range of the data, Fig. 9(i) summarizes the intervals by showing the average width across the range of observed peak-to-peak values. Over the seven *ex vivo* eyes, the width of the calibration confidence interval is approximately 5% to 10%, with a median of 8.4%. In other words, an estimate of the hydration loss derived from peak-to-peak measurements is associated with a 95% calibration confidence interval that is about 8.4 percentage points wide. Values of the coefficients of determination, regression slopes, and calibration confidence interval widths are provided for each eye sample in Table 2.

## 5. Discussion

This work serves as an initial validation of a compact, high-speed, tabletop scanner system for direct, *in vivo* hydration mapping using THz spectral imaging. Our scanner is capable of full spectral imaging of corneal-scale targets in less than a second, by integration of an ECOPS-based THz-TDS system, which captures 100-ps waveforms at 2 kHz, into a novel spherical scanning architecture. With a 10×10 grid covering 40°×40° FOV, the system captures a full image in 0.8 s, substantially reducing sensitivity to involuntary motion compared to multi-second scans. Additional automatic motion compensation based on the measured first-surface TOA corrects both lateral and axial misalignment, further improving the robustness to motion.

Using both a contact lens phantom model and *ex vivo* eye globe samples, we observed a strong linear correlation between the reflected THz signal and bulk hydration calculated from gravimetric measurements. However, both categories of samples also showed evolving spatial patterns in hydration maps. This heterogeneity highlights the limitations of hydration metrics derived from point measurements. The differences between the reflectivity and optical thickness maps of the contact lens phantom further suggest that indirectly estimating hydration based on an assumed relationship with the optical thickness is insufficient, especially when the thickness is measured at only a single location.

The heterogeneity in the THz images also demonstrates the limitations of bulk measurements for the assessment of hydration. Gravimetric measurement provided a reliable, continuous ground truth for the contact-lens phantom, where the sample’s water content is uniformly distributed at the start of the experiments and mass loss is a direct proxy for hydration. Still, even though the entirety of the evaporative loss can be attributed to the contact lens itself, the THz images reveal a complex local variation in hydration that is not captured in the bulk measurements. These limitations become more pronounced in the *ex vivo* eye experiments, where gravimetric measurements represent the average hydration of the entire eye globe, rather than the cornea alone. In other words, the hydration loss is spatially non-uniform across the ocular surface in *ex vivo* samples. For example, although the tissue had not completely dried by the end of the experiments, the exposed tissue at the front of the eyes was more desiccated than the areas covered by the holder. This difference suggests that the change in the corneal tissue hydration may be underestimated by the measurements of mass loss alone. Despite these limitations, gravimetric measurements provide a practical reference standard for hydration assessment in phantoms and *ex vivo* samples. In future phases of this project, *in vivo* gravimetric measurement will not be feasible. Therefore, clinical validation of the THz hydration signal will require comparison against an independent, spatially resolved thickness reference, such as OCT-based pachymetry at matched locations.

These findings highlight the potential of THz imaging for corneal measurement, in which spatial information about both sample geometry and reflectivity measurements are captured simultaneously. Furthermore, signal-processing techniques such as sparse deconvolution can enhance the sensitivity to these aspects, as we demonstrated for the measurements of the contact lens. Biological corneas, however, exhibit lower reflectivity than the phantom, and extending the application of these techniques will require improved time-windowing, deconvolution and model-based separation methods to resolve the multiple closely spaced interfaces (e.g., tear film, epithelium, and stroma). Translating the system to *in vivo* measurement will additionally require further streamlined alignment and faster processing for point-of-care results, with a focus on integrating real-time tracking.

## 6. Conclusions

We demonstrated a compact THz-TDS system for rapid spectroscopic imaging of spherical surfaces, such as corneas. By combining 2-kHz waveform acquisition, high-speed custom phase-front-matched beam steering, and time-of-arrival-based automatic alignment, the system acquired 40° × 40° images in 0.8 s. Further, it can compensate for eye motion causing lateral and axial sample displacements between scans. Measurements of a drying contact-lens phantom and *ex vivo* porcine eye globes showed that THz reflection amplitude and optical thickness are sensitive to hydration-related changes and can reveal spatial variations that are not represented by bulk gravimetric measurements. Sparse deconvolution further enabled separation of closely spaced interface reflections in the contact-lens phantom. These findings show the ability of our THz scanner to resolve hydration gradient maps of the cornea in less than a second. Future work will focus on real-time motion tracking, improved separation of corneal-layer reflections, quantitative calibration of local hydration, and *in vivo* validation testing.

## Figures and Tables

**Fig. 1.**
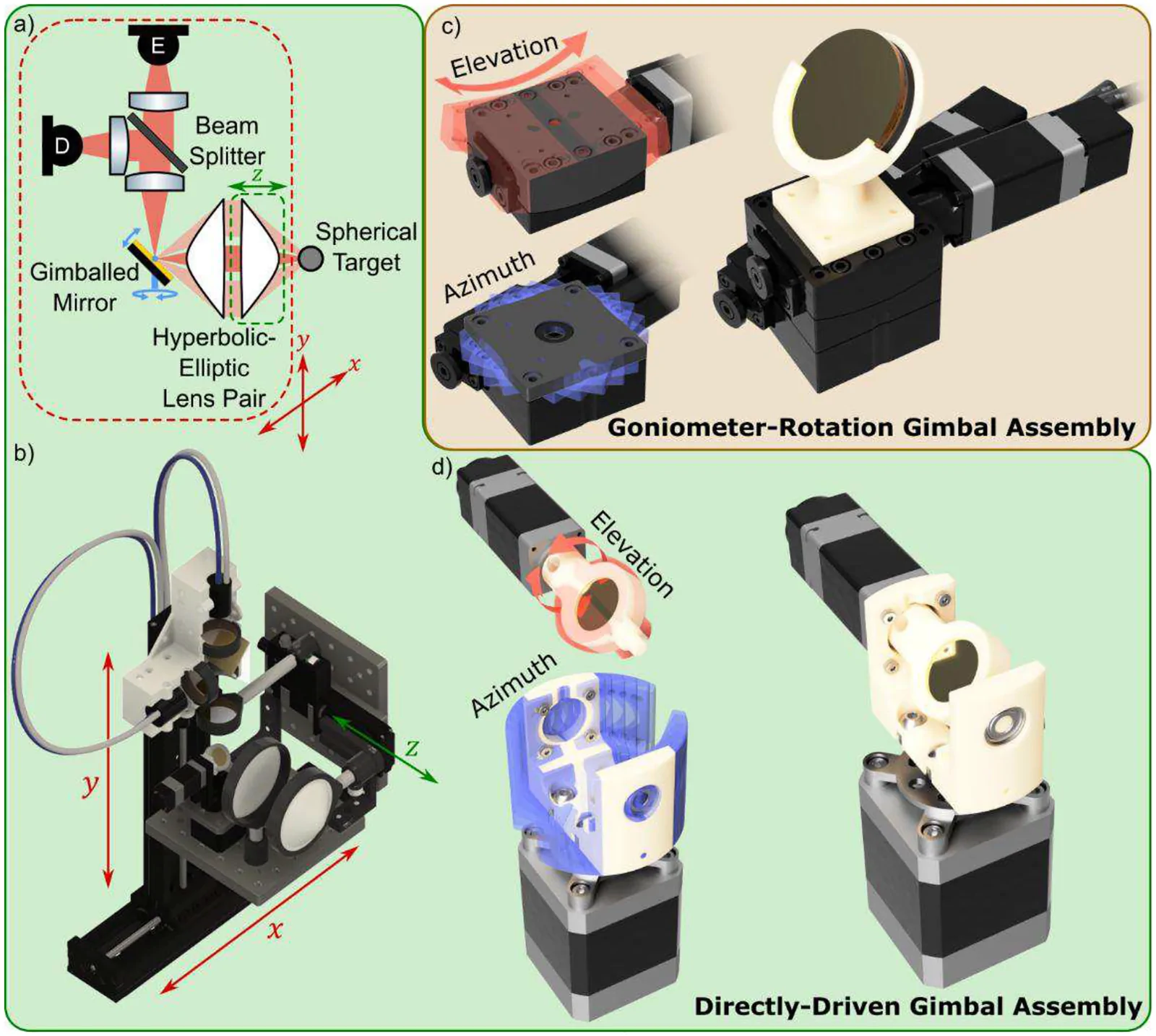
(a) Schematic of the THz corneal scanning system. (b) Rendering showing motorized stages used for automatic focusing and motion compensation. The dashed green box in (a) illustrates translation of only the final lens along the z axis, whereas the dashed red box indicates translation of the entire scanner along the x and y axes. (c) Illustration of the previous goniometer-rotation gimbal assembly showing the limited scanning range of the goniometer stage (red overlays) used for the elevation axis, and rotation axis of the rotation stage (blue overlays) used for the azimuthal axis. (d) Directly-driven gimbal design used in the current system. The motions of the two axes (elevation in red, azimuthal in blue), follow the same axes as the previous assembly shown in (c) but are now driven directly.

**Fig. 2.**
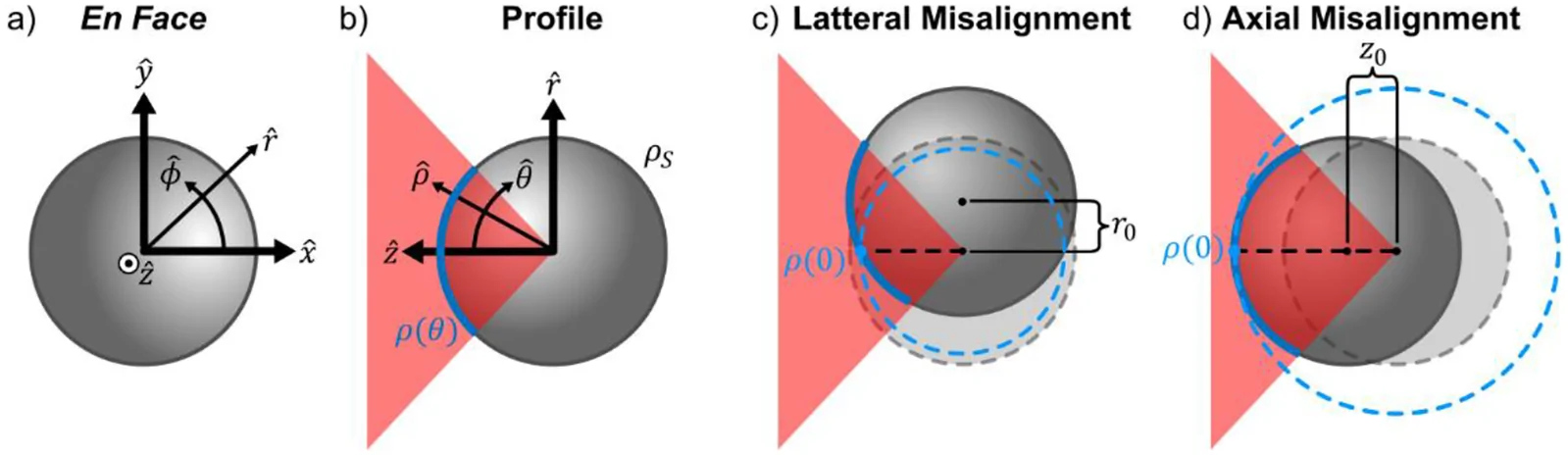
Coordinate systems and effects of misalignment. (a) *En face* and (b) profile coordinate systems used to define the scanning space. The red cone indicates the scanner FOV. (c, d) Effects of lateral and axial misalignment by offset distances of $r_{0}$ and $z_{0}$, respectively, on the measured surface profile Δρ. The dashed gray circles indicate the position of a well aligned sphere. The solid blue curves show the measured surface profile while the dashed blue lines indicate the surface of a properly aligned sphere with radius $\rho_{S}=\rho (\theta =0)$, against which the surface profile is measured.

**Fig. 3.**
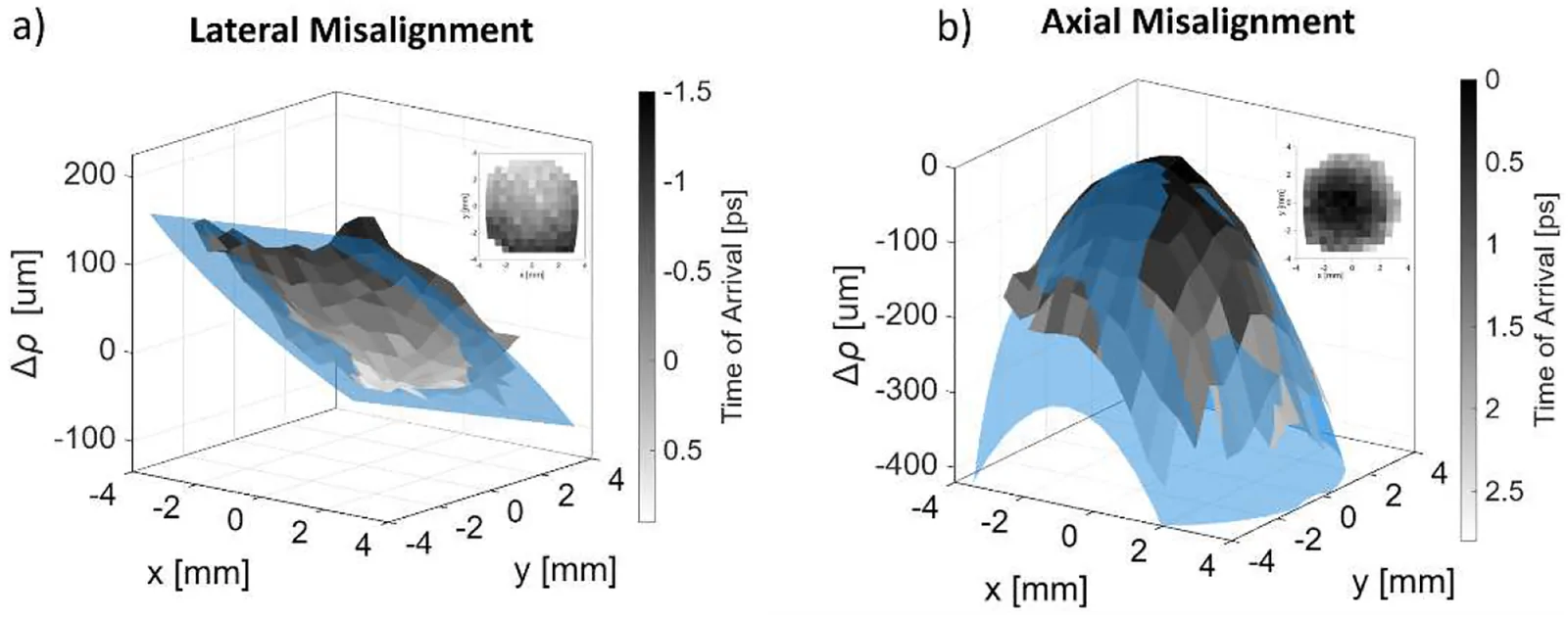
Measured surface profile Δρ for an 8 mm radius metal sphere with (a) lateral and (b) axial offsets. The inset image in each subfigure shows top-down (*en face*) views. The blue surface in (a) represents the plane fitting used to separate x and y components of the lateral shift while the blue surface in (b) shows the full quadratic fit.

**Fig. 4.**
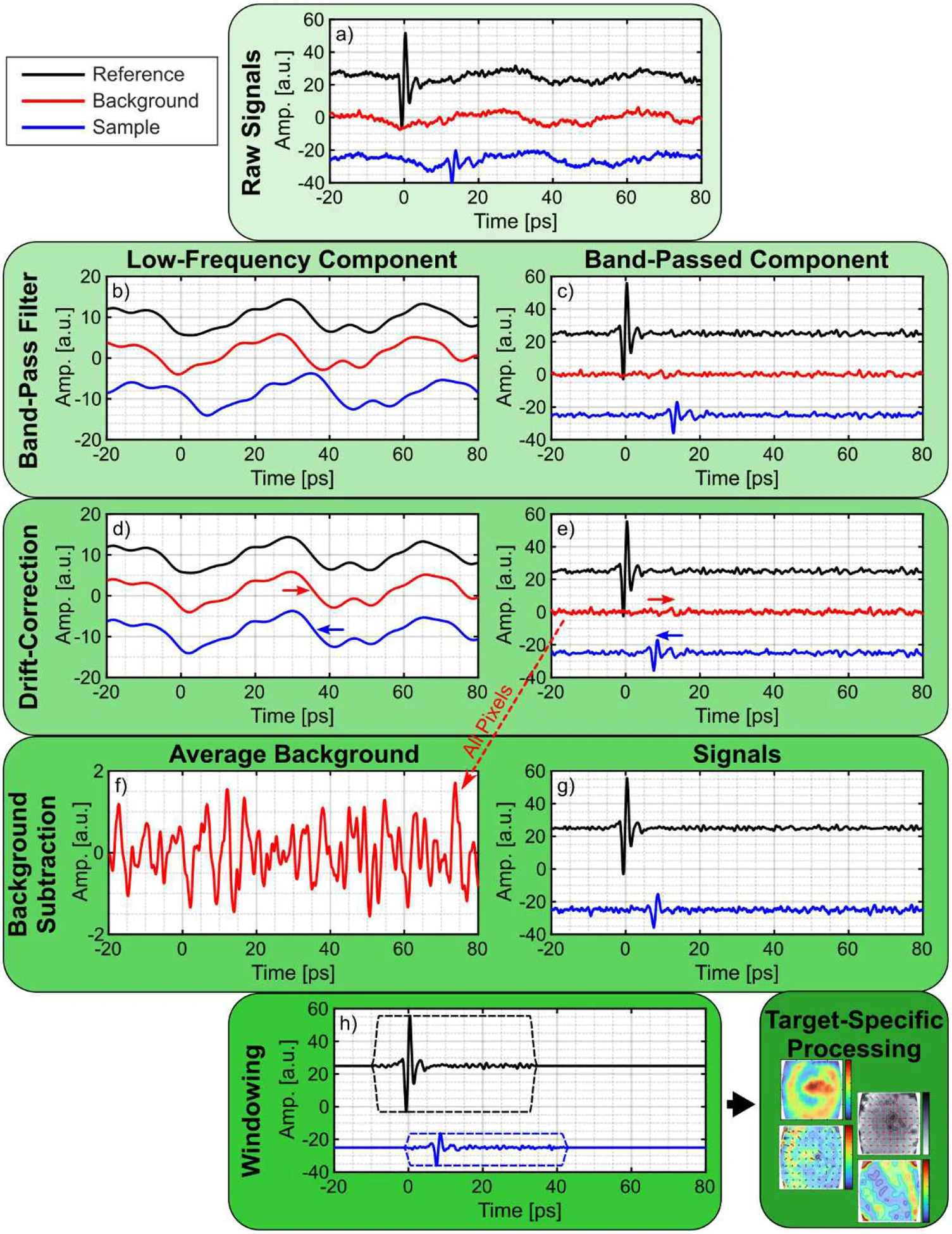
System background signal removal steps. (a) Raw traces acquired for a reference sphere (black), the background (red, measured without a target in place), and from the sample (blue). After filtering, (b) the low-frequency baseline and (c) mid-frequency signal components are separated while the high frequency noise is discarded. (d) The system drift is estimated by aligning the low frequency components and (e) the same shift is applied to the mid-frequency traces. (f) The remaining background signal within the passband is calculated by averaging the traces from all background image pixels. (g) This average background is subtracted from the reference and sample traces. (h) The signals are windowed to isolate the reflections in preparation for target-specific processing. The magnitude of the drifts in (a)-(c) have been exaggerated to more clearly illustrate the procedure.

**Fig. 5.**
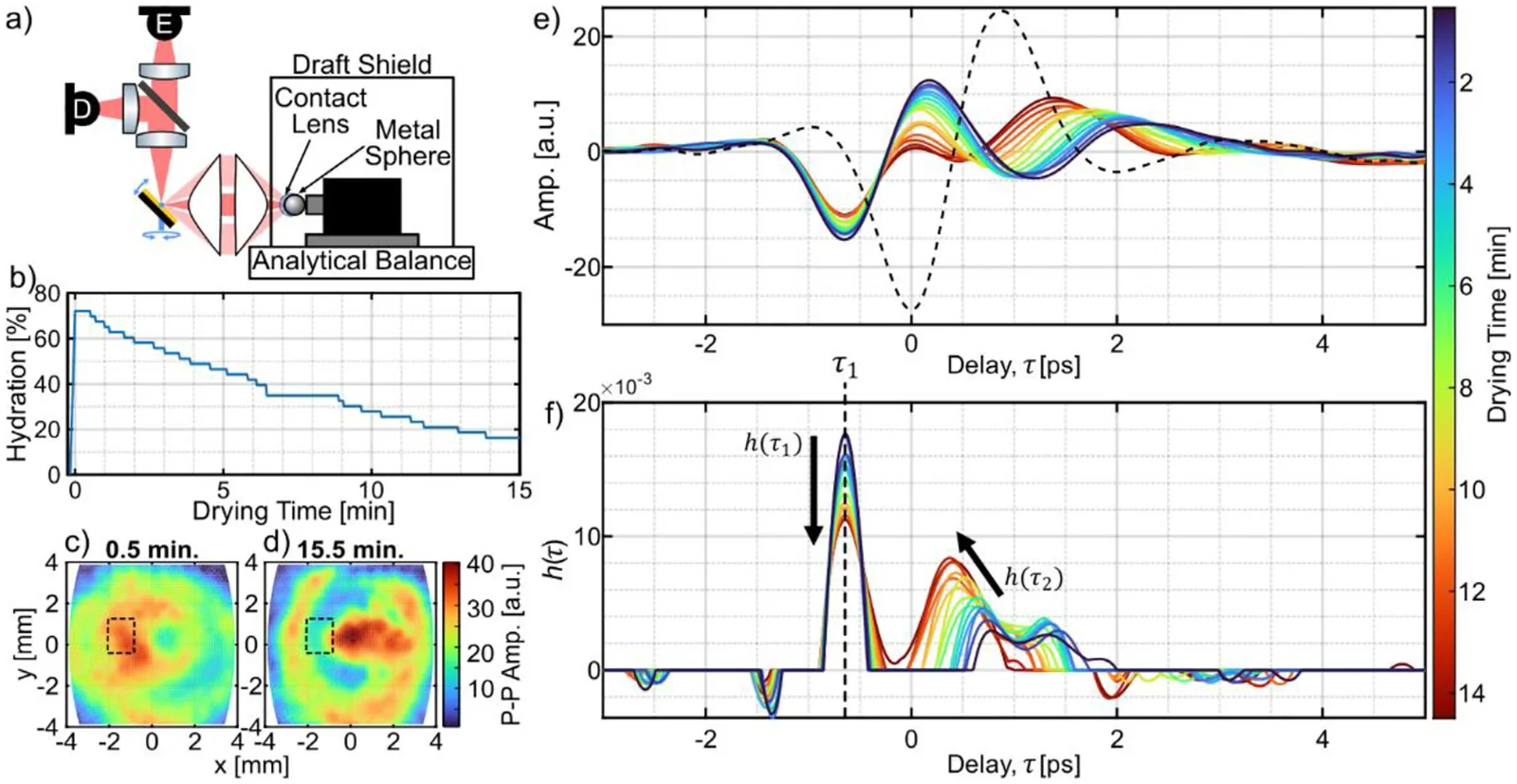
(a) Corneal phantom (contact lens) measurement setup. The sphere was placed within the draft shield of the analytical balance, while the side facing the scanner was left open to avoid beam obstruction. (b) Bulk hydration over the first 15 min of the drying experiment. (c)-(d) Images of the peak-to-peak amplitude (between −2 and −0.2 ps) acquired during contact-lens drying; the dashed box indicates the region of interest used for analysis. The influence of the metal reflection is clearly seen in the center of the image despite the windowing function. (e) Mean reflected time-domain waveforms from the region of interest over time with the black dashed line showing the signal from the bare metal ball. (f) The corresponding impulse responses calculated from sparse deconvolution. The line colors in (e)-(f) indicate acquisition time.

**Fig. 6.**
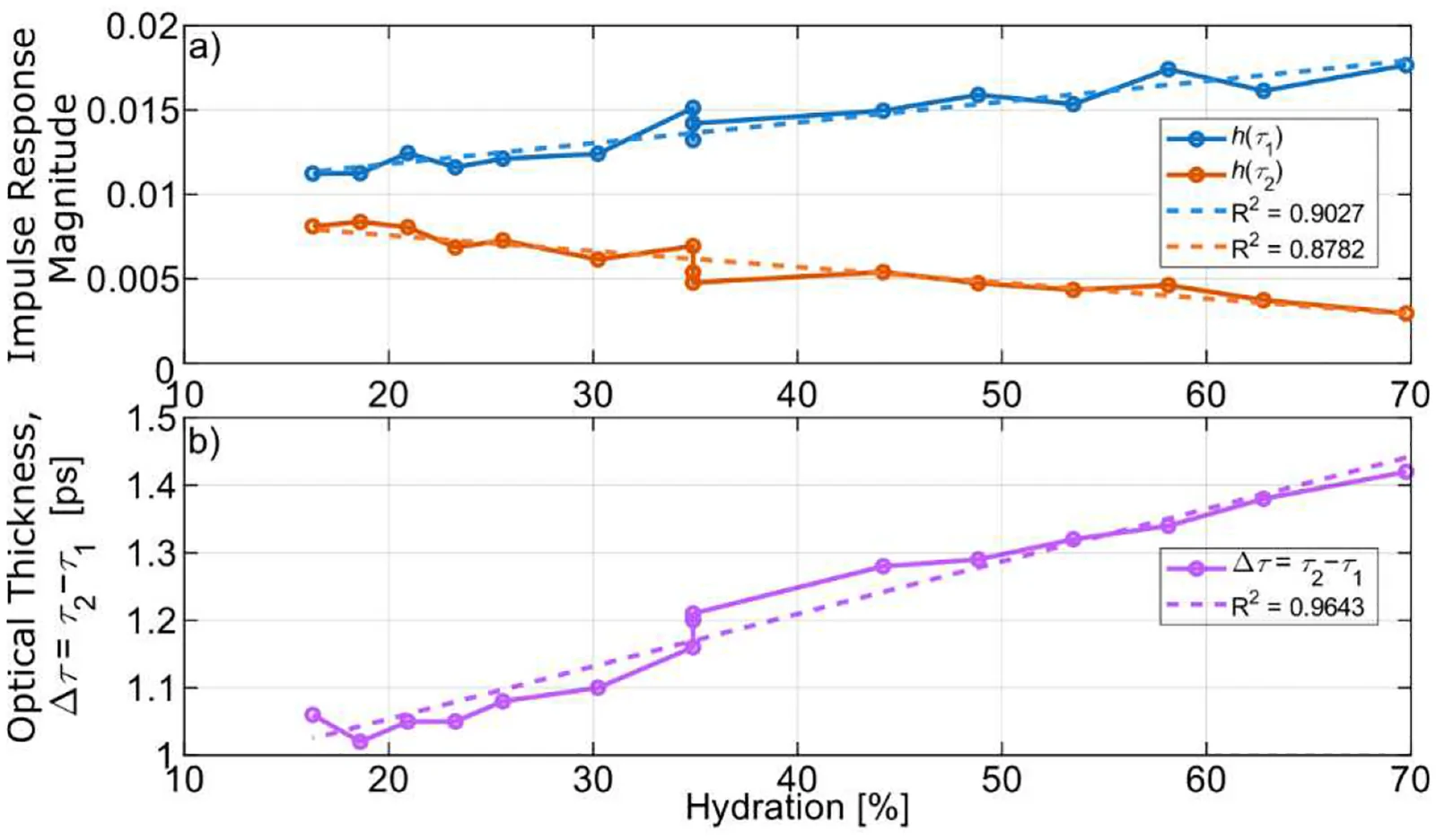
Variations in the impulse response function of the drying contact lens. (a) Plot of impulse response magnitude of peaks at $\tau_{1}$ (blue) and $\tau_{2}$ (red) averaged over the ROI shown in Fig. 5 as a function of the hydration (%). The dashed lines show a linear fit to the data. (b) Change in the round-trip optical thickness, Δτ, of the lens as a function of hydration and dashed line indicating the linear fit.

**Fig. 7.**
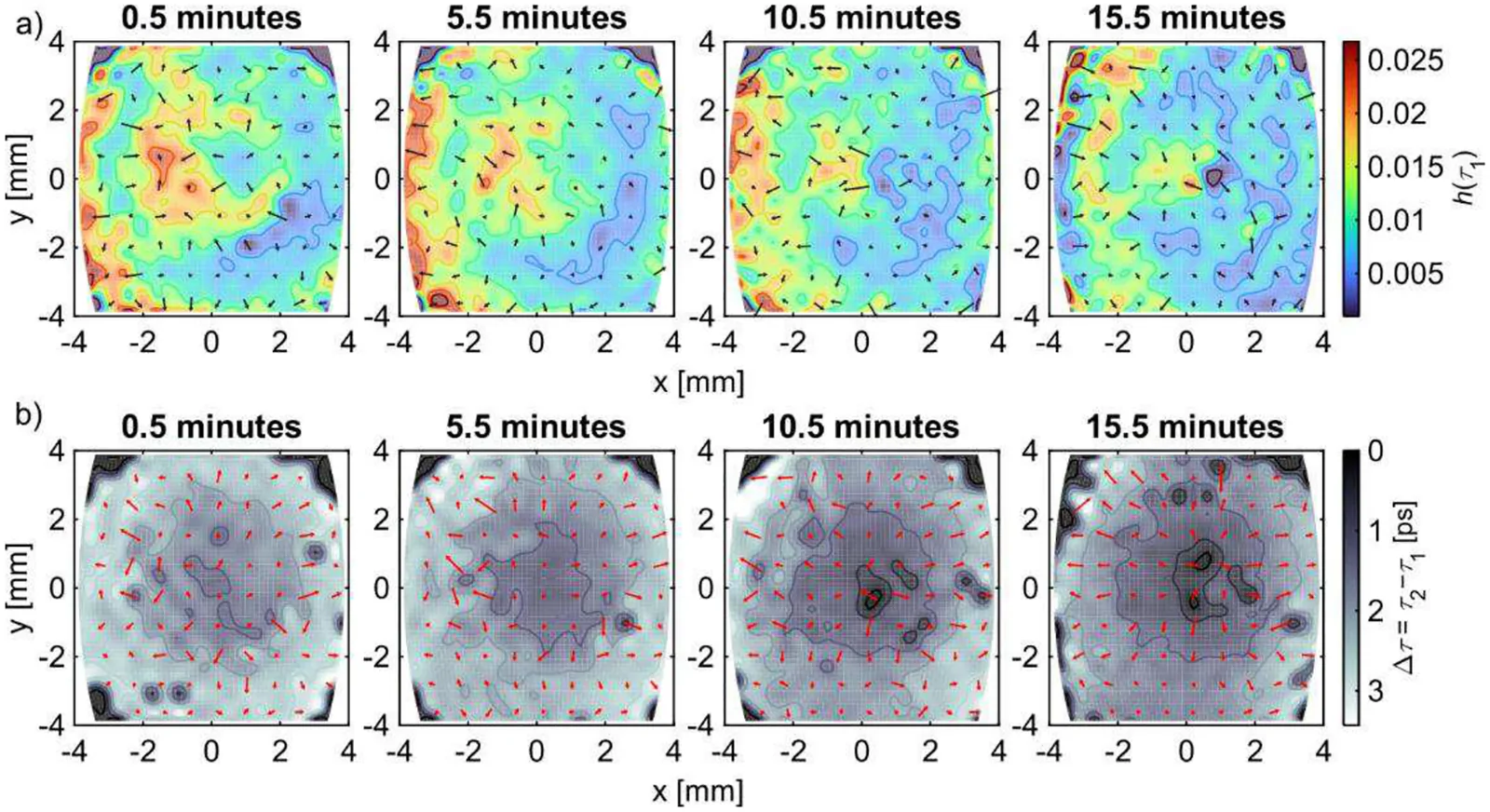
Spatial and longitudinal evolution of the impulse response function of the drying contact lens. (a) Spatial maps of the first reflection peak as the lens dried. (b) Round-trip optical thickness, Δτ, of the contact lens calculated from the difference of the two impulse peaks, $\tau_{2}-\tau_{1}$. Arrows indicate the spatial gradient of the corresponding images, showing the different evolution of their spatial patterns as the contact dried.

**Fig. 8.**
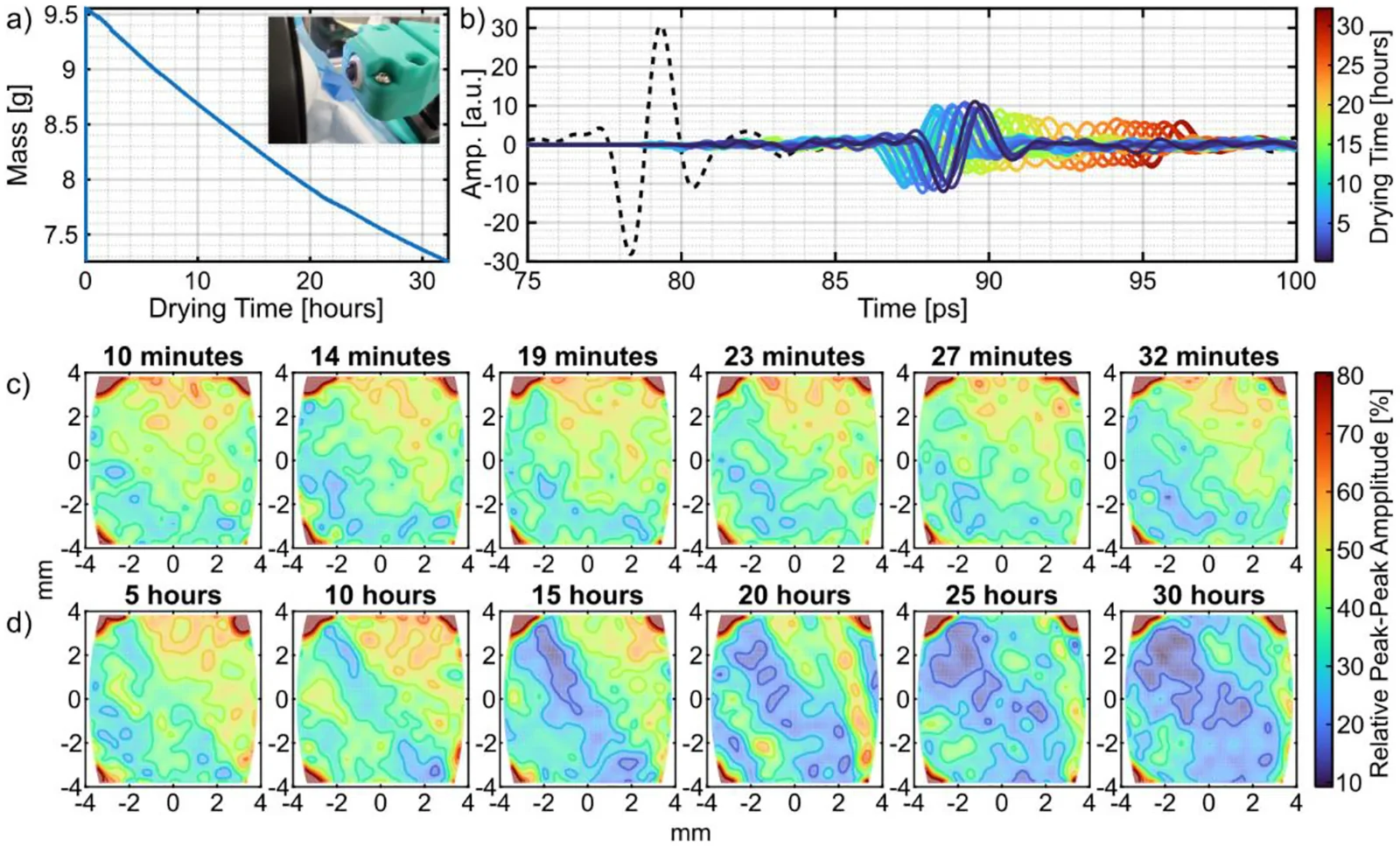
Measurement of drying *ex vivo* eye globe sample G. (a) Mass of globe measured over time as water evaporated from the tissue. The inset photo shows an eye globe mounted in the sample holder during measurements. The scanner objective lens at the left of the image was automatically aligned to the sample. (b) THz time-domain traces averaged over an ROI at the center of the images. Line color indicates acquisition time. (c-d) THz-derived hydration maps of the eye at different time-points during the drying period. The series in (c) shows early time-points approximately 4–5 minutes apart, while the series in (d) shows images over five-hour increments through the end of the experiment.

**Fig. 9.**
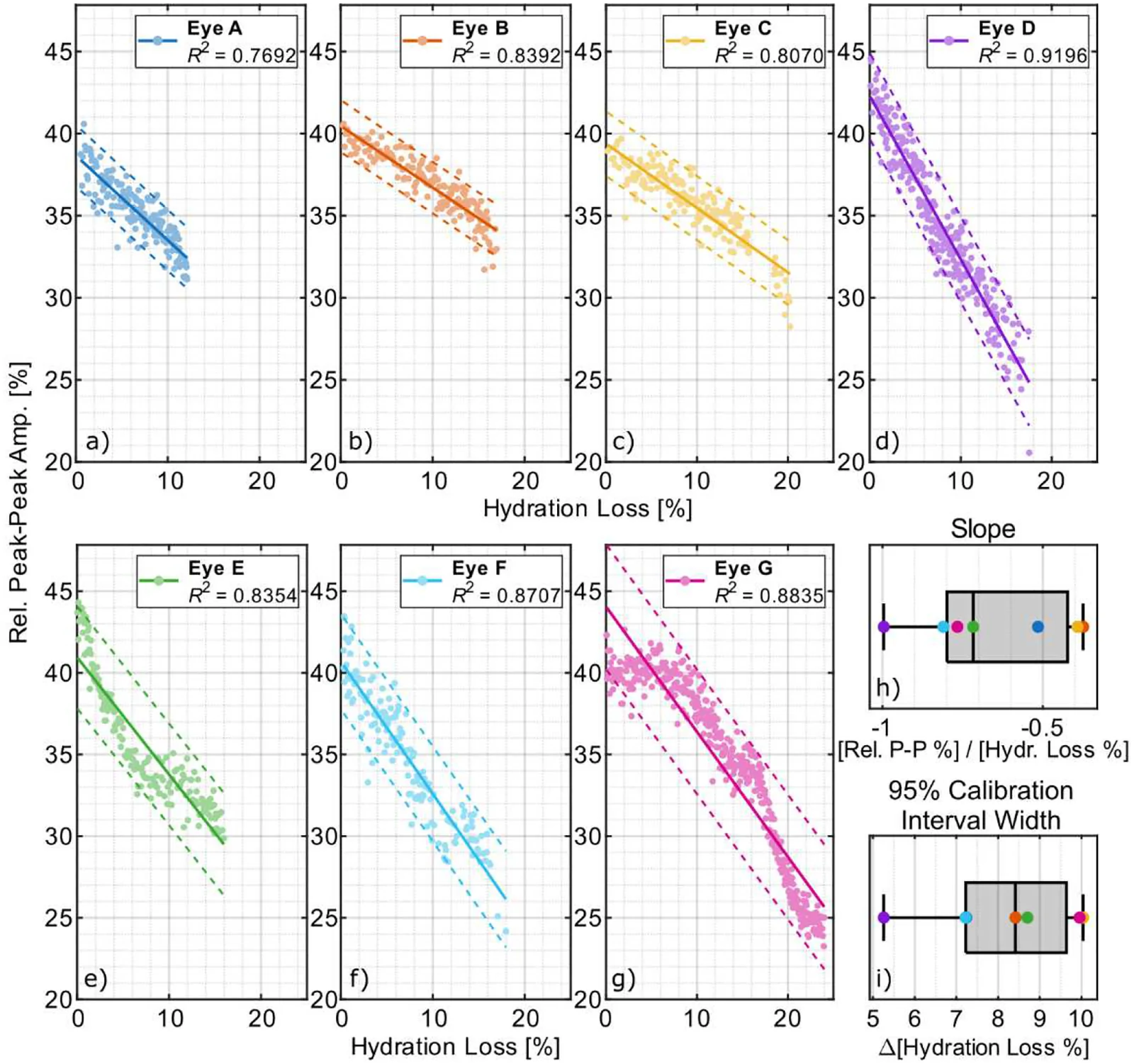
Comparisons of the peak-to-peak amplitudes to the mass loss percentage for each sample. (a)-(g) show fits (solid lines) and 95% prediction interval (dashed lines) for each *ex vivo* globe sample. (h) Distribution of the slopes of each fit. (i) Distribution of the 95% calibration confidence interval widths calculated as the average width of the prediction band for a specific peak-to-peak amplitude. The markers in (h) and (i) represent the values from each eye.

**Table 1.** Summary of changes in mass and hydration during THz measurement for each *ex vivo* globe

Sample	Lab Relative Humidity [%]	Measurement duration [hh:mm]	Initial Mass [g]	Final Mass [g]	Final Hydration Loss [%]
**A**	27	12:50	9.97	8.77	12.1
**B**	36^a^	12:13	8.35	6.94	17.0
**C**	22^a^	15:06	8.07	6.44	20.2
**D**	48	25:58	11.46	9.45	17.5
**E**	43	14:52	9.81	8.24	16.0
**F**	42	21:05	9.71	7.97	18.0
**G**	48	32:12	9.55	7.26	24.0

a Limited data due to sensor failure

**Table 2.** THz sensitivity metrics for each *ex vivo* globe

Sample	Regression ${\text{R}}^{2}$	Regression Slope [P-P %]/[Hydration Loss %]	Calibration Confidence Interval 𝚫 [Hydration Loss %]
**A**	0.7692	−0.515	7.24
**B**	0.8392	−0.374	8.41
**C**	0.8070	−0.392	10.0
**D**	0.9196	−0.996	5.26
**E**	0.8354	0.717	8.70
**F**	0.8707	−0.810	7.22
**G**	0.8835	−0.766	10.0

## Data Availability

Data underlying the results presented in this paper are not publicly available at this time but may be obtained from the authors upon reasonable request.
